# Organic fertilizer enhances microbial functional genes related to nitrogen and phosphorus cycling in rubber tree (*Hevea brasiliensis*) rhizosphere

**DOI:** 10.3389/fmicb.2026.1833968

**Published:** 2026-04-29

**Authors:** Shunjun Geng, Xiaoling Shi, Qianghao Zhang, Jifen Yang, Chunxia Yang, Liping Yang

**Affiliations:** 1 Yunnan Key Laboratory of Sustainable Utilization Research on Rubber Tree, Xishuangbanna, China; 2 Yunnan Institute of Tropical Crops, Xishuangbanna, China

**Keywords:** functional gene, *Hevea brasiliensis*, metagenomics, nitrogen cycle, organic fertilizer, phosphorus cycle

## Abstract

**Introduction:**

Nitrogen (N) and phosphorus (P) are the essential nutrient for rubber growth. However, the effect of organic fertilizer application on soil microbial communities and functional genes related to N and P cycling in rubber plantation are unclear.

**Methods:**

A field trial was established in a rubber plantation with two treatments: organic fertilizer (OF) and an unfertilized control (CK). In this study, we used metagenomics analysis to examine the structural and functional alterations in the microbial community within the rhizospheric soil of rubber when organic fertilizers were applied.

**Results:**

Results showed that compared with the CK treatments, the OF treatment significantly increased soil organic matter (SOM), total nitrogen (TN), total phosphorus (TP), alkali-hydrolyzable nitrogen (AN), and available phosphorus (AP) contents. Taxonomic analysis revealed that OF treatment significantly enriched the phyla Pseudomonadota and Myxococcota, and the genera *Pseudolabrys* and *Gaiella*. At the functional level, organic fertilization significantly up-regulated key genes associated with N cycling, including organic N metabolism (*gltB*), N transport (*nrtA, nrtB, nrtC*), denitrification (*norB, nosZ*), nitrification (*nxrB*), and dissimilatory nitrate reduction (*napA, napC*). Regarding the P cycle, organic fertilization leads to the downregulation of the high-affinity phosphate transporter gene *pstS* and the concurrent upregulation of genes governing organic P mineralization (*phnA*, *phoN*), regulation (*phoB*), polyphosphate synthesis (*ppk1*), and polyphosphate degradation (*spoT*, *relA*). The variation partitioning analysis (VPA) results indicated that pH, SOM, and nitrogen nutrients (comprising TN and AN) explained 71.52% of the variation in the abundance of nitrogen-cycling functional genes, while pH, SOM, and phosphorus nutrients (comprising TP and AP) explained 64.95% of the variation in the abundance of phosphorus-cycling functional genes.

**Conclusion:**

In summary, the application of organic fertilizer reshapes soil microbial communities and enhances the functional potential for nitrogen (N) and phosphorus (P) cycling. Our study provides a mechanistic basis for developing sustainable nutrient management strategies to optimize N and P bioavailability in tropical rubber agroecosystems.

## 1 Introduction

Natural rubber, derived primarily from *Hevea brasiliensis*, is an indispensable industrial commodity, with its cultivation concentrated in the tropical regions characterized by highly weathered, acidic soils. The productivity and sustainability of rubber plantations are intrinsically linked to the soil’s capacity to cycle essential nutrients and maintain its ecological functions. Among these nutrients, nitrogen (N) and phosphorus (P) are primary constraints on plant growth and development.

The intensive management of agricultural systems, including rubber plantations, has historically relied on substantial N fertilizer inputs. While this approach can enhance yields in the short term, it is often associated with low nitrogen use efficiency (NUE) and a cascade of adverse environmental consequences, including aquatic eutrophication, increased emissions of greenhouse gasses (e.g., nitrous oxide), and accelerated soil acidification (Galloway et al., 2008; Guo et al., 2010). Recent studies continue to highlight the global scale of these impacts (Mirzaee and Nafchi, 2025; Sun et al., 2021). Concurrently, phosphorus poses a dual challenge. A significant proportion of the world’s agricultural soils, approximately 40%, are deficient in biologically available phosphorus, limiting crop productivity (Zhu et al., 2018). Paradoxically, the long-term and excessive application of phosphate fertilizers to overcome this deficiency has led to the accumulation of legacy P in soils, which becomes a chronic source of non-point source pollution, driving freshwater eutrophication and increasing the risk of P loss from terrestrial ecosystems (Demay et al., 2023). Consequently, a paradigm shift is required in nutrient management—one that moves beyond mere application to focus on improving the efficiency of N use and unlocking the large, yet largely inaccessible, reserves of P in the soil. Deciphering the soil microbial mechanisms that govern these nutrient transformations is critical for developing sustainable agricultural practices that maintain productivity while mitigating environmental harm.

Soil N and P cycling are fundamental biogeochemical processes that underpin soil fertility and ecosystem functioning, directly supporting the growth and activity of both plants and microorganisms (Hu et al., 2022). The soil N cycle is a complex network of transformations—including organic N metabolism, nitrification, denitrification, N_2_-fixation, and assimilatory (ANRA) and dissimilatory (DNRA) nitrate reduction—that are primarily mediated by microorganisms harboring specific functional genes (Kuypers et al., 2018; Zhang et al., 2019). Similarly, the soil P cycle is governed by microbial communities through processes such as inorganic P solubilization and organic P mineralization. These transformations are facilitated by key functional genes, including those encoding for polyphosphate kinases (*ppk1*), phosphatases (*phoD*, *phoA*), phosphate transporters (*pst*), and two-component regulatory systems (*phoB*, *phoR*) (Liu et al., 2018).

Accumulating evidence indicates that organic fertilization not only improves soil physicochemical properties and stimulates microbial activity (Bhardwaj et al., 2014; García-Orenes et al., 2016; Hsiao et al., 2025) but also profoundly reshapes microbial community composition and the abundance of functional genes driving nutrient cycling. For instance, in the black soil region of Northeast China, Hu et al. (2022) demonstrated that organic fertilizer application significantly increased the abundance of nitrate reduction genes (*nasD* and *napA*), thereby enhancing both assimilatory (ANRA) and dissimilatory (DNRA) nitrate reduction pathways. Similarly, long-term organic amendment in vineyards was shown to substantially elevate the abundance of the nitrogen fixation gene *nifH* and key denitrification genes (*nirS, nirK,* and *nosZ*) (Pereg et al., 2018). A meta-analysis by Wu et al. (2025) further corroborated these findings, reporting that organic fertilization enhances soil microbial biomass phosphorus, acid phosphatase activity, and the abundance and diversity of *phoD* and *phoC* genes, which are pivotal for organic phosphorus mineralization.

The impact of organic fertilizers on phosphorus cycling is particularly pronounced in nutrient-limited soils. In a calcareous soil under a sweet potato–wheat rotation system, Pereg et al. (2018) observed that organic fertilizer application significantly increased soil available phosphorus and alkaline phosphatase activity. This was attributed to a shift in the microbial community, characterized by an enrichment of phosphate-solubilizing bacteria (e.g., *Acidiphilium, Panacagrimonas*, and *Beijerinckia*) and the concurrent upregulation of key functional genes involved in phosphorus acquisition, namely *pqqC* and *phoD*. These findings collectively underscore the critical role of organic amendments in modulating microbially mediated nutrient cycles, thereby contributing to improved soil fertility and ecosystem sustainability.

Despite this growing body of evidence, a systematic understanding of how organic fertilization drives microbial community succession, coordinates the expression of functional genes, and mediates interactions with environmental factors in the context of tropical rubber plantations remains elusive. To address this knowledge gap, we integrated metagenomic sequencing with comprehensive soil physicochemical analyses. Our specific objectives were to: (1) quantify the extent to which organic fertilization restructures the soil microbial community in rubber plantations; (2) delineate the response patterns of key functional genes governing nitrogen and phosphorus cycling; and (3) resolve the coupled relationships among microbial taxa, functional gene assemblages, and key environmental variables. By elucidating these microbial-ecological mechanisms, this study aims to provide a mechanistic understanding of how organic fertilization regulates soil N and P cycling in rubber plantations, thereby offering a theoretical foundation for developing sustainable nutrient management strategies and improving soil fertility in this critical agroecosystem.

## 2 Materials and methods

### 2.1 Experimental site

The field experiment was conducted at the rubber plantation experimental station of the Yunnan Institute of Tropical Crops Science, Yunnan Province, China (22°1′N, 100°46′E). This site is characterized by a tropical monsoon climate. The rubber clone *Hevea brasiliensis* Yunyan 77-4, planted in 2012 and tapped since 2021, was used in this study. Trees were spaced at 2.5 m × 9.0 m. The initial physiocochemical properties of the soil at 0–20 cm depth were as follows: pH 4.67, soil organic matter (SOM) 27.28 g/kg, total nitrogen (TN) 1.77 g/kg, total phosphorus (TP) 0.32 g/kg, total potassium (TK) 22.42 g/kg, alkali-hydrolyzable nitrogen (AN) 110.13 mg/kg, available phosphorus (AP) 15.84 mg/kg and available potassium (AK) 108.69 mg/kg.

### 2.2 Experimental design

The experiment was established in September 2022 using a randomized complete block design. Two treatments were implemented: organic fertilizer application (OF; 5 kg per tree) and an unfertilized control (CK). Each treatment was replicated four times, with each replicate plot consisting of 10 trees, resulting in a total of 80 trees. For the OF treatment, fertilizer trenches (100 cm long × 40 cm wide × 40 cm deep) were excavated between adjacent trees. The organic fertilizer, contained 1.00% N, 1.12% P₂O₅, and 1.20% K₂O, was applied evenly into the trench and thoroughly with the soil before backfilling.

### 2.3 Sample collection and determination

In September 2023, soil samples from the rhizosphere were gathered from the fertilization furrows. Four replicate samples were collected per treatment. Each sample was homogenized and divided into two subsamples. A portion of the sample was sieved through a 2 mm sieve, quickly frozen, and kept at −80 °C for later DNA extraction. The other subsample underwent air drying and sieving before being used to analyze soil physicochemical properties. The pH of the soil was determined using a pH meter in a soil-to-water suspension with a ratio of 1:2.5 (w/v). The soil organic matter (SOM) content was determined using the potassium dichromate external heating method. Total nitrogen (TN) was measured using the Kjeldahl technique. For total phosphorus (TP), soil samples were digested with concentrated H₂SO₄ and HClO₄, and the P concentration in the digest was analyzed using an AA3 continuous-flow analyzer. Alkali-hydrolyzable nitrogen (AN) was assessed through the method of alkaline hydrolysis diffusion. Available phosphorus (AP) was measured with an AA3 continuous-flow analyzer following extraction with NH₄F solution.

### 2.4 Soil metagenomic sequencing

Using the FastDNA Spin Kit for Soil (MP Biomedicals, Solon, OH, United States), total genomic DNA was extracted from 0.5 grams of each soil sample following the manufacturer’s guidelines. DNA concentration was quantified using a TBS-380 fluorometer (Turner Biosystems, Sunnyvale, CA, United States), and DNA integrity was assessed by 1% (w/v) agarose gel electrophoresis. Purified DNA from each sample was sent to Majorbio Bio-Pharm Technology Co., Ltd. (Shanghai, China) for shotgun metagenomic sequencing on the Illumina NovaSeq platform (PE150).

Fastp (v0.23.0) was employed to process raw sequencing reads by trimming adapters and filtering out low-quality reads; reads with a length less than 50 bp after trimming or with an average Phred quality score below 20 were discarded. All samples’ high-quality reads were *de novo* co-assembled using MEGAHIT (v1.1.2), and contigs that were 300 bp or longer were preserved for subsequent analysis. Using Prodigal (v2.6.3) (Hyatt et al., 2010) and MetaGene (Noguchi et al., 2006), open reading frames (ORFs) were identified from the assembled contigs. Predicted genes with nucleotide lengths ≥100 bp were retained and translated into amino acid sequences using EMBOSS (v6.6.0). A non-redundant gene catalog was constructed by clustering the predicted gene sequences using CD-HIT (v4.6.1) with a threshold of 90% sequence identity and 90% coverage; the longest sequence from each cluster was selected as the representative sequence. To determine the abundance of each gene, high-quality reads from each sample were aligned to the non-redundant gene catalog using SOAPaligner (v2.21), allowing no more than five mismatches and requiring a minimum identity of 95%.

For taxonomic assignment, representative sequences of the non-redundant gene catalog were aligned to the NCBI NR database using DIAMOND (v0.8.35) with an E-value cutoff of 1 × 10^−5^. To annotate functions, representative sequences were compared to the Kyoto Encyclopedia of Genes and Genomes (KEGG) database^1^ using BLASTP (E-value ≤1 × 10^−5^). Genes involved in nitrogen and phosphorus cycling were identified based on their KEGG Orthology (KO) assignments. Abundances of genes associated with these cycles were normalized to reads per kilobase per million mapped reads (RPKM) and used for subsequent analyses of functional composition, differential abundance, and correlations.

### 2.5 Statistical analysis

The statistical analysis was conducted with SPSS 19.0, while figures were produced using Origin version 2024. Using the Kruskal–Wallis nonparametric test, variations in soil microbial community composition and functional gene abundances between the organic fertilizer (OF) and control (CK) treatments were assessed, followed by *post hoc* pairwise comparisons with *p*-values adjusted using the Benjamini–Hochberg false discovery rate (FDR) correction. Differences with adjusted *p* < 0.05 were considered statistically significant.

Redundancy analysis (RDA) was employed to explore the relationships between soil physicochemical properties and both the soil microbial community composition (at the phylum level) and the relative abundances of nitrogen and phosphorus cycling functional genes. Ordination analysis was performed using CANOCO (v5.1.2). Prior to RDA, microbial and functional gene relative abundance data were Hellinger-transformed using the decostand function. Soil physicochemical property data were log10-transformed and then Z-score standardized to achieve normality and comparable scales. Statistical significance for the RDA models was tested using Monte Carlo permutation tests with 999 permutations.

To quantify the relative contributions of soil physicochemical properties to the variations in functional gene abundances, variation partitioning analysis (VPA) was performed. For nitrogen-cycling genes, the explanatory variables were partitioned into three independent matrices: nitrogen nutrients (comprising TN and AN), pH, and SOM. Analogously, for phosphorus-cycling genes, the soil physicochemical property drivers were categorized into phosphorus nutrients (comprising TP and AP), pH, and SOM. All VPA procedures were conducted using the *vegan* package in R (Version 2.4.3).

## 3 Results

### 3.1 Effects of organic fertilizer application on soil chemical properties

The application of organic fertilizer (OF) substantially improved the soil nutrient status compared to the unfertilized control (CK) (Table 1). Specifically, the OF treatment resulted in significantly higher levels of SOM, TN, TP, AN, and AP (*p* < 0.05). In contrast, soil pH remained statistically unchanged between the two treatments, indicating that organic fertilization enhanced nutrient availability without altering soil acidity in the short term.

**Table 1:** Basic soil physicochemical properties of under different treatments (means ± SE).

Treatment	pH	SOM	AN	AP	TN	TP
		(g/kg)	(mg/kg)	(mg/kg)	(g/kg)	(g/kg)
CK	4.60 ± 0.11a	29.29 ± 2.27b	111.70 ± 13.42b	12.19 ± 1.71b	1.63 ± 0.10b	0.37 ± 0.03b
OF	4.63 ± 0.06a	56.28 ± 4.60a	167.08 ± 13.98a	93.79 ± 11.03a	1.93 ± 0.13a	0.94 ± 0.11a

Different letters in the same column indicate significant differences among treatments at *p* < 0.05. CK, no fertilization; OF, organic fertilizer. SOM, soil organic matter; AN, alkali-hydrolyzable nitrogen; AP, available phosphorus; TN, total nitrogen; TP, total phosphorus.

### 3.2 Shifts in soil microbial community composition following organic fertilization

**Figure 1:**
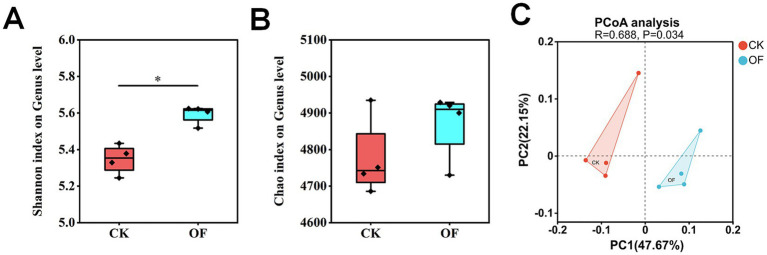
Soil microbial community diversity indices and principal coordinates analysis (PCoA) ordination under different fertilization treatments. **(A)** Shannon diversity index. **(B)** Chao1 richness index. **(C)** PCoA of microbial community composition at the genus level. CK, no fertilization; OF, organic fertilizer. Significance levels: * *p* < 0.05; ns, not significant.

Organic fertilizer application induced substantial shifts in soil microbial community structure (Figure 1C). While microbial richness, as estimated by the number of observed operational taxonomic units (Genus), was significantly higher in the OF compared to the CK treatment (*p* < 0.05; Figure 1A), no significant difference in overall microbial diversity (e.g., Shannon index) was detected between treatments (Figure 1B).

**Figure 2:**
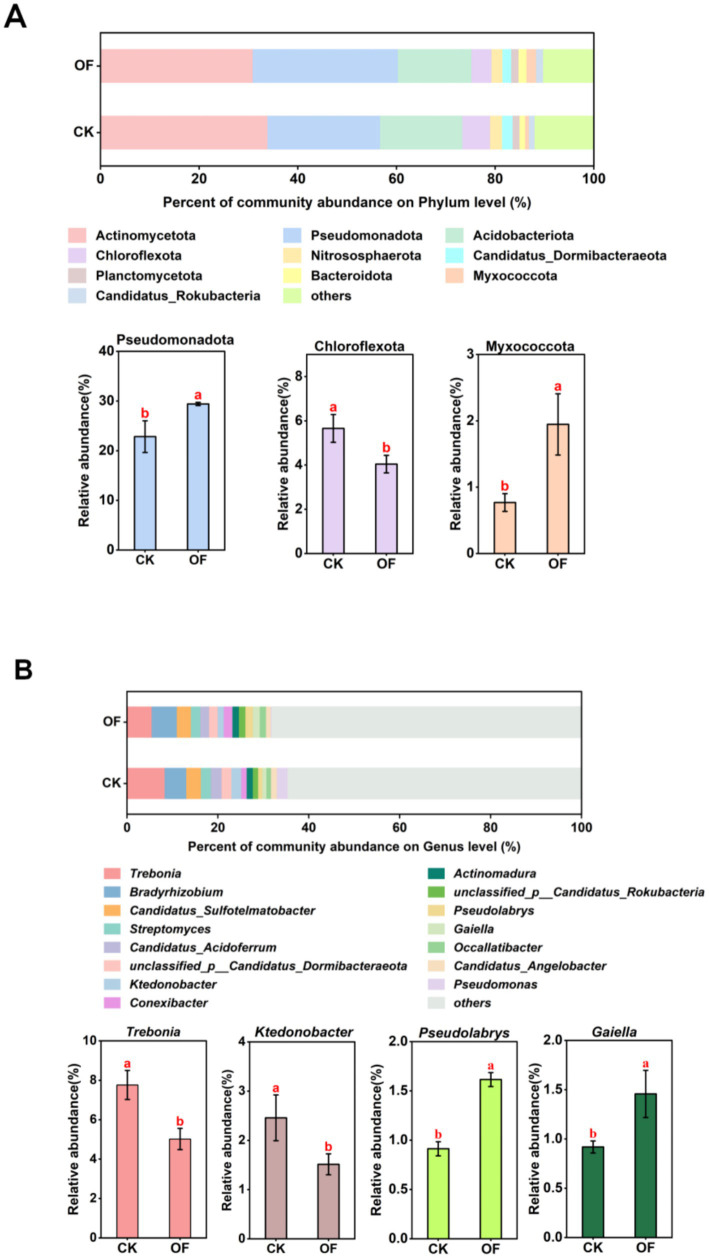
Effects of organic fertilization on soil microbial community composition. Relative abundance of dominant microbial communities at the phylum level **(A)** and at the genus level **(B)**. Different letters indicate significant differences among treatments at *p* < 0.05. CK, no fertilization; OF, organic fertilizer.

At the phylum level, the microbial communities were consistently dominated by Actinomycetota (30.91–33.89%), Pseudomonadota (22.83–29.40%), and Acidobacteriota (14.94–16.66%) across both treatments (Figure 2A). However, organic fertilization significantly altered the relative abundances of specific phyla. Notably, the OF treatment resulted in a significant enrichment of Pseudomonadota and Myxococcota (*p* < 0.05), while the relative abundance of Chloroflexota was significantly reduced (*p* < 0.05) compared to the CK.

At the genus level, *Trebonia* (30.91–33.89%), *Bradyrhizobium* (4.85–5.57%), and *Candidatus* Sulfotelmatobacter (2.85–3.04%) were the predominant taxa (Figure 2B). Organic fertilization led to significant shifts in key genera, with *Pseudolabrys* and *Gaiella* being significantly enriched (*p* < 0.05) and *Trebonia* and *Ktedonobacter* being significantly depleted (*p* < 0.05) in the OF relative to the CK treatment.

### 3.3 Response of nitrogen-cycle functional genes to organic fertilization

**Figure 3:**
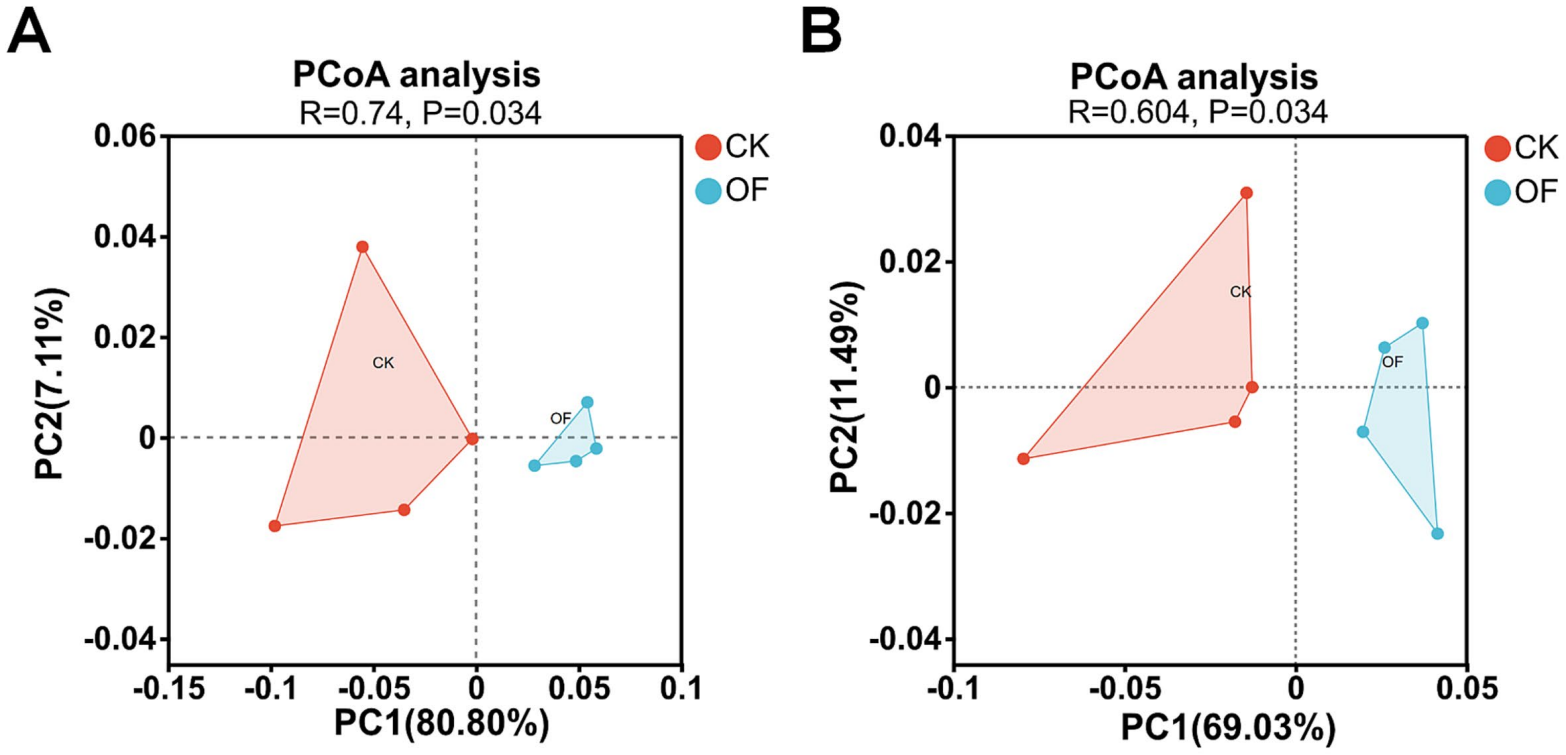
Principal coordinates analysis (PCoA) of nitrogen- **(A)** and phosphorus-cycling **(B)** functional genes based on the Bray–Curtis dissimilarity. CK, no fertilization; OF, organic fertilizer. Percentages on axes indicate the proportion of total variance explained by each principal coordinate.

Principal coordinate analysis (PCoA) based on the abundance of N-cycling genes revealed a clear separation between the OF and CK treatments along the first principal coordinate (PC1), which explained 80.80% of the total variation (Figure 3A). This pronounced divergence indicates that organic fertilization profoundly reshaped the functional potential of the soil N-cycling microbiome.

**Figure 4:**
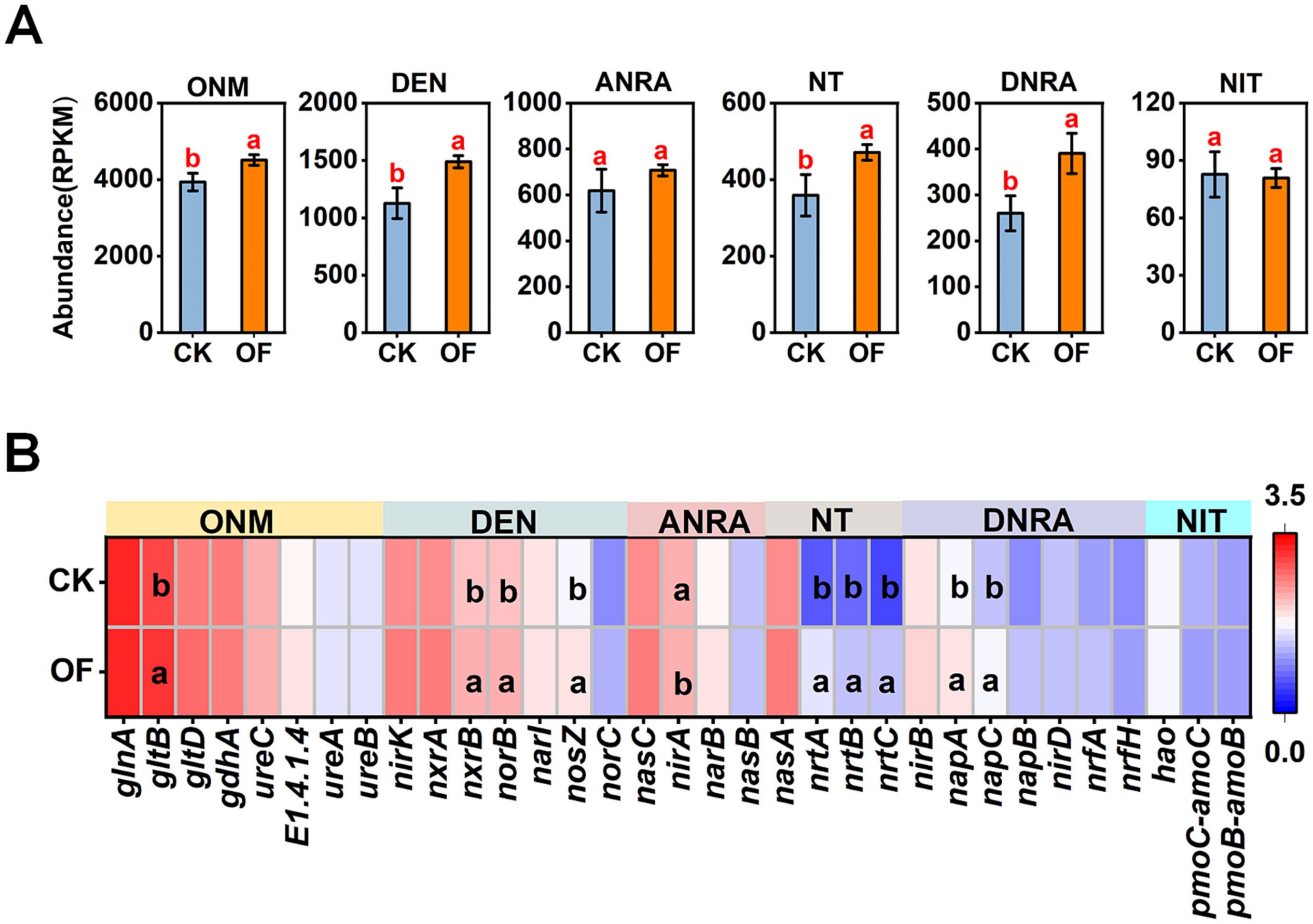
Variations in relative abundance of nitrogen-cycling genes under different treatments at the **(A)** functional level and **(B)** gene level. Different letters indicate significant differences among treatments at *p* < 0.05. ONM, organic N metabolism; DEN, denitrification; ANRA, assimilatory nitrogen (N) reduction; NT, N transport; DNRA, dissimilatory N reduction; NIT, nitrification; CK, no fertilization; OF, organic fertilizer.

Differential abundance analysis of N-cycling pathways revealed that the OF treatment significantly enhanced the genetic potential for several key processes compared to CK (*p* < 0.05). Specifically, genes associated with organic nitrogen metabolism, denitrification, nitrogen transport, and dissimilatory nitrate reduction to ammonium (DNRA) were significantly enriched in the OF-treated soils (Figure 4A).

At the individual gene level, the OF treatment led to significant increases in the abundance of genes encoding key enzymes involved in these pathways (*p* < 0.05; Figure 4B). These included genes involved in organic N metabolism (*gltB*), denitrification (*norB, nosZ*), N transport (*nrtA, nrtB, nrtC*), dissimilatory nitrate reduction (*napA, napC*), and nitrification (*nxrB*). Conversely, the abundance of *nirA*, a gene involved in assimilatory nitrate reduction, was significantly reduced in the OF treatment compared to CK (*p* < 0.05).

### 3.4 Response of phosphorus-cycle functional genes to organic fertilization

The abundance of phosphorus (P)-cycling genes demonstrated a clear separation between the OF and CK treatments along the first principal coordinate (PC1), which accounted for 69.03% of the total variation in P metabolism (Figure 3B). This distinct clustering indicates that organic fertilization substantially altered the functional capacity of the soil P-cycling microbiome.

**Figure 5:**
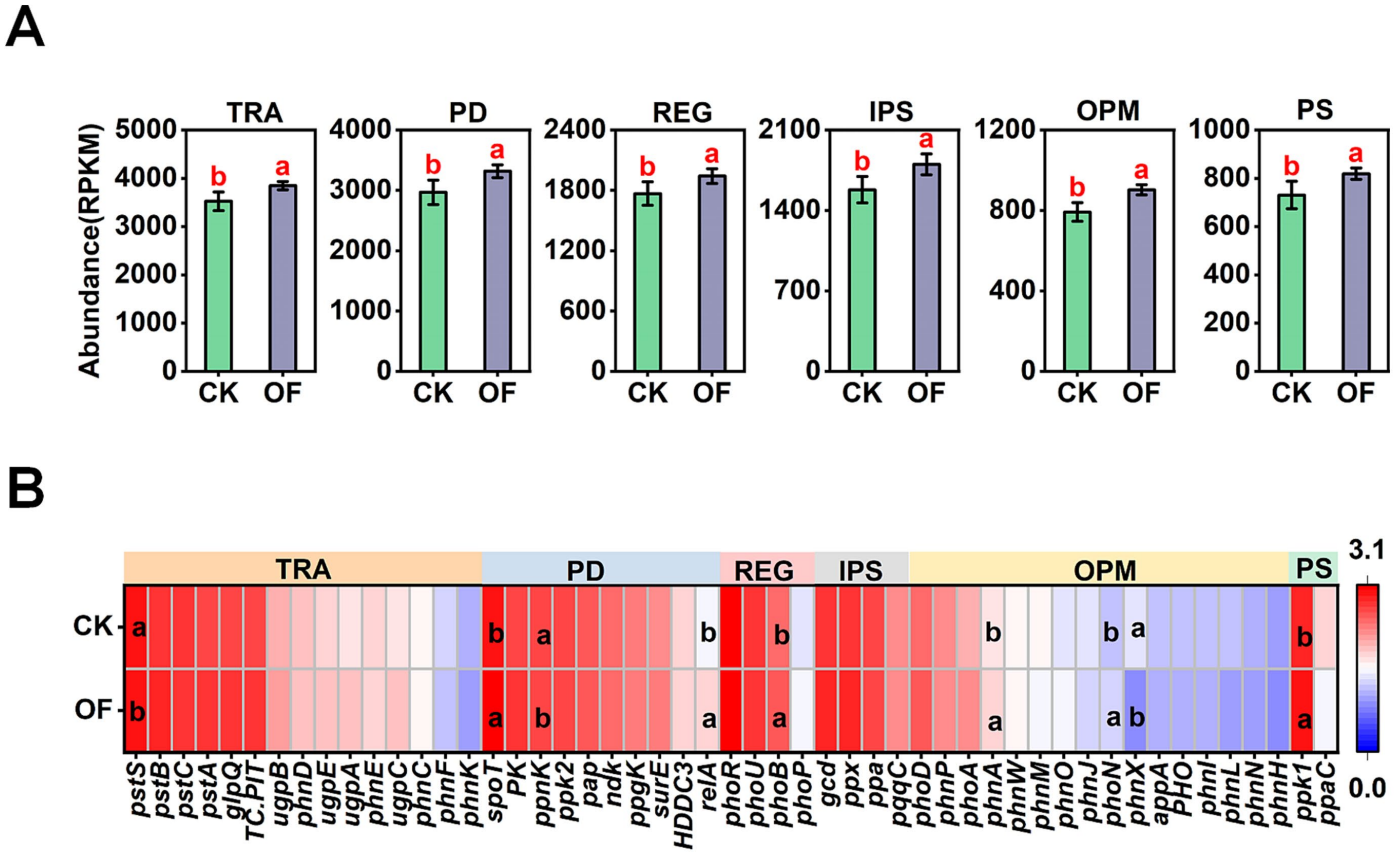
Variation in relative abundance of phosphorus-cycling genes in different treatments at the **(A)** functional level and **(B)** gene level. Different letters indicate significant differences among treatments at *p* < 0.05 level. TRA, transporters; PD, polyphosphate degradation; REG, regulatory; IPS, inorganic phosphorus (P) solubilization; OPM, organic P mineralization; PS, polyphosphate synthesis; CK, no fertilization; OF, organic fertilizer.

Analysis of P-cycling pathways revealed that the OF treatment significantly enhanced the genetic potential for multiple key processes compared to CK (*p* < 0.05). Specifically, genes associated with P transport, polyphosphate degradation, regulatory systems, inorganic P solubilization, organic P mineralization, and polyphosphate synthesis were all significantly enriched in the OF-treated soils (Figure 5A).

At the individual gene level, organic fertilization resulted in significant differential abundance of several key functional genes (*p* < 0.05; Figure 5B). For instance, the OF treatment significantly increased the relative abundances of genes involved in stringent response regulation (*spoT*, *relA*), the two-component regulatory system (*phoB*), phosphonate degradation (*phnA*), nonspecific acid phosphatase (*phoN*), and polyphosphate kinase (*ppk1*). In contrast, the abundances of genes encoding polyphosphate/ATP NAD kinase (*ppnK*), the phosphate transport system substrate-binding protein (*pstS*), and phosphonoacetaldehyde hydrolase (*phnX*) were significantly reduced in the OF compared to the CK treatment.

### 3.5 Relationships among soil microbial communities, functional genes, and soil environmental factors

To disentangle the complex interactions between soil properties and microbial functional potential, we integrated redundancy analysis (RDA) with Spearman’s rank correlation coefficients.

#### 3.5.1 Drivers of microbial community composition

**Figure 6:**
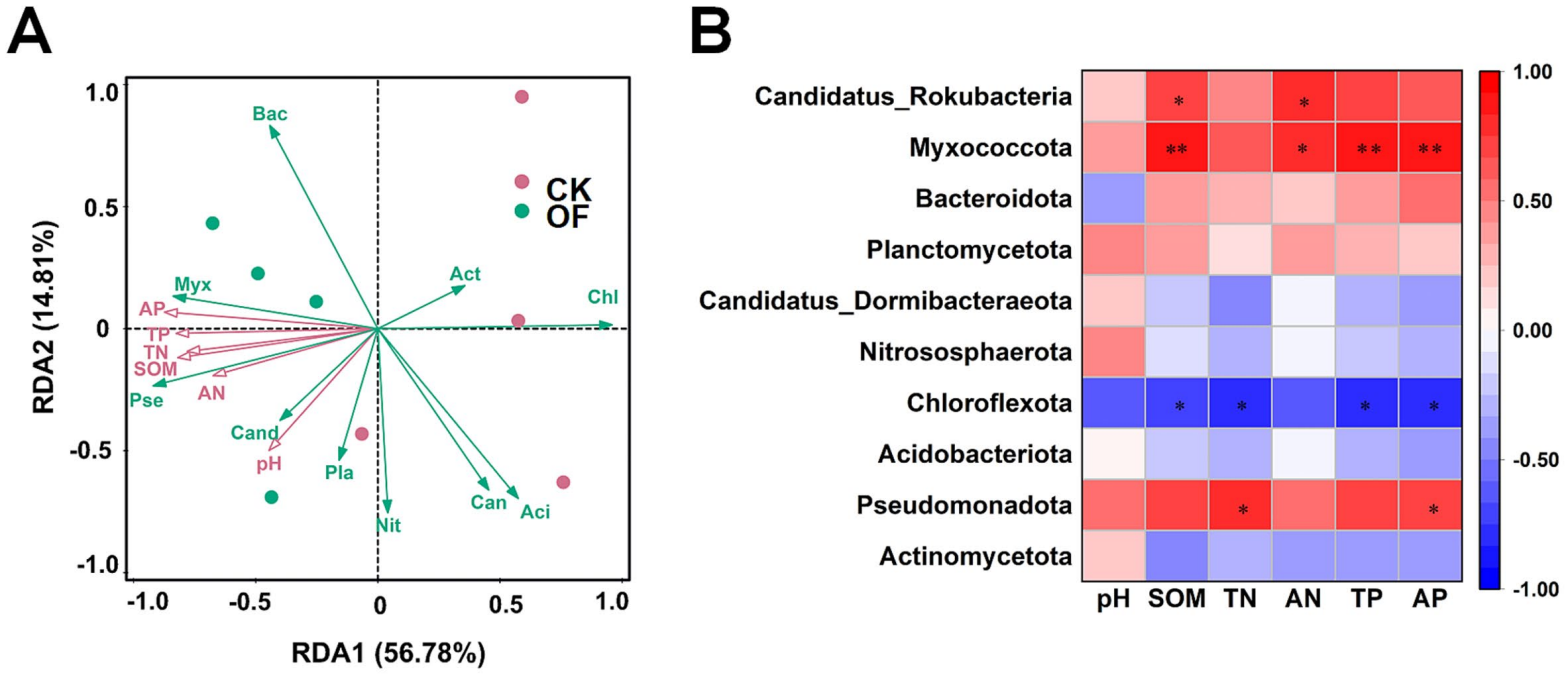
Relationships between microbial communities and soil physicochemical properties. **(A)** Redundancy analysis (RDA) ordination plot illustrating the effects of soil properties on microbial community structure at the phylum level plot. **(B)** Spearman’s rank correlation heatmap showing correlations between the relative abundances of dominant phyla and soil physicochemical parameters. Actinomycetota: Act, Pseudomonadota: Pse, Acidobacteriota: Aci, Chloroflexota: Chl, Nitrososphaerota: Nit, Candidatus_Dormibacteraeota: Can, Planctomycetota: Pla, Bacteroidota: Bac, Myxococcota: Myx, Candidatus_Rokubacteria: Cand. CK, no fertilization; OF, organic fertilizer; SOM, soil organic matter; TN, total nitrogen; TP, total phosphorus; AN, alkaline-hydrolyzable nitrogen; AP, available phosphorus. Significant correlations are indicated as follows: * *p* < 0.05; ** *p* < 0.01.

The redundancy analysis was employed to elucidate the relationships between soil physicochemical properties and microbial community structure at the phylum level (Figure 6A). The first two RDA axes collectively explained 71.59% of the total variation in community composition (axis 1: 56.78%; axis 2: 14.81%). The Spearman’s correlation analysis further resolved the specific responses of key phyla to soil properties (Figure 6B). The relative abundance of Pseudomonadota was significantly positively correlated with TN and AP (*p* < 0.05). Myxococcota abundance exhibited significant positive correlations with SOM, AN, TP, and AP (*p* < 0.05). Conversely, Chloroflexota abundance showed significant negative correlations with SOM, TN, TP, and AP (*p* < 0.05), indicating a contrasting ecological response to nutrient enrichment.

#### 3.5.2 Environmental drivers of nitrogen-cycling genes

**Figure 7:**
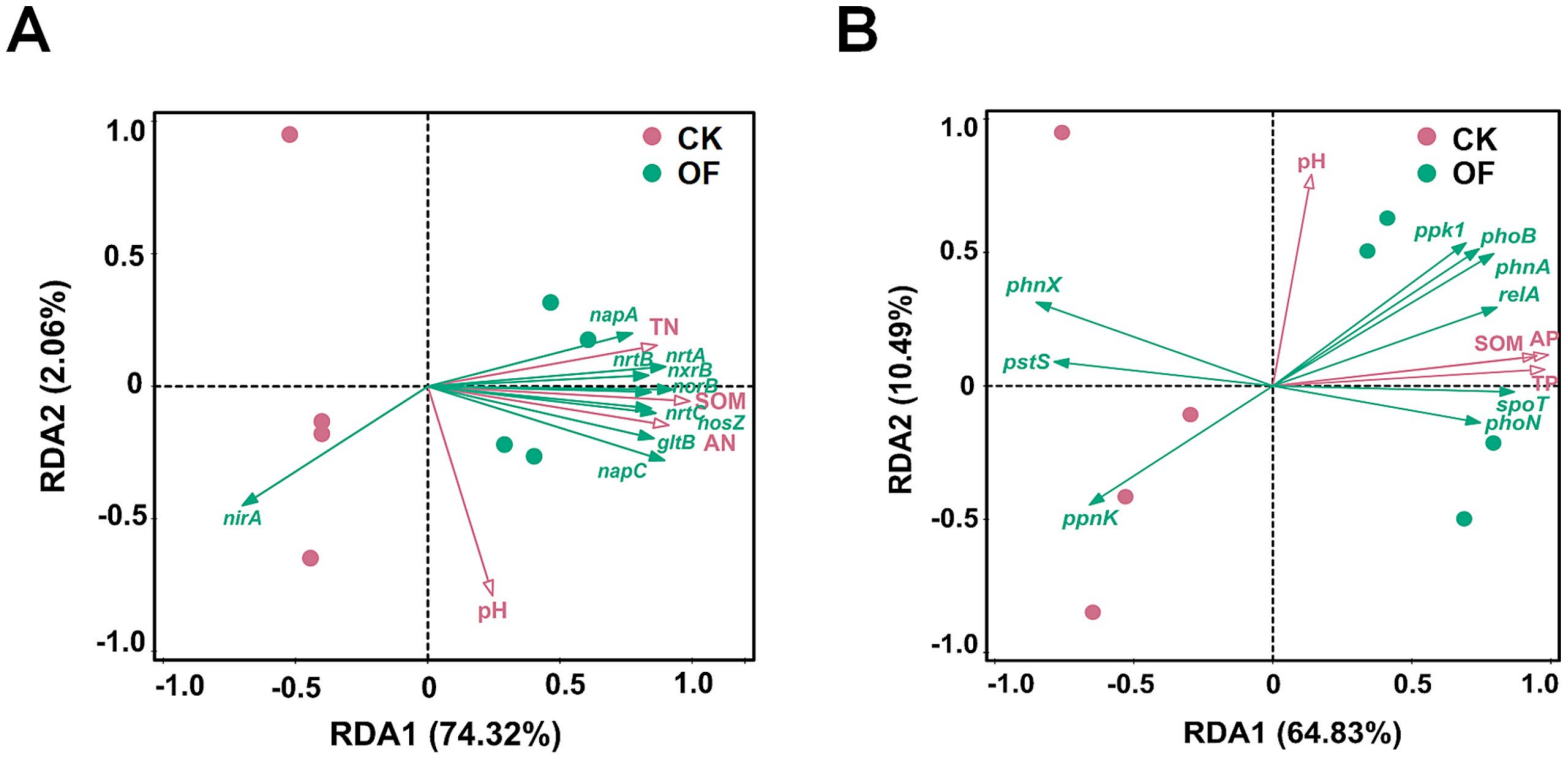
Redundancy analysis (RDA) of soil **(A)** nitrogen-cycling and **(B)** phosphorus-cycling functional genes and physicochemical properties. **(A)** RDA ordination plot showing the relationship between nitrogen-cycling functional genes and soil physicochemical properties. **(B)** RDA ordination plot showing the relationship between phosphorus-cycling functional genes and soil physicochemical properties. Arrows indicate the direction and strength of environmental variables in explaining the variation in functional gene composition. SOM, soil organic matter; TN, total nitrogen; TP, total phosphorus; AN, alkaline-hydrolyzable nitrogen; AP, available phosphorus; CK, no fertilization; OF, organic fertilizer.

The RDA was also applied to assess the influence of soil properties on the profile of N-cycling functional genes (Figure 7A). The first two axes accounted for 76.38% of the explained variance (axis 1: 74.32%; axis 2: 2.06%). In addition, variation partitioning analysis (VPA) indicated that pH, SOM, and nitrogen nutrients collectively accounted for 71.52% of the variation in the abundance of N-cycling functional genes. Specifically, pH explained 10.62% of the total variation, followed by SOM (7.21%). Notably, the shared explanatory power of SOM and nitrogen nutrients reached 87.78%.

**Figure 8:**
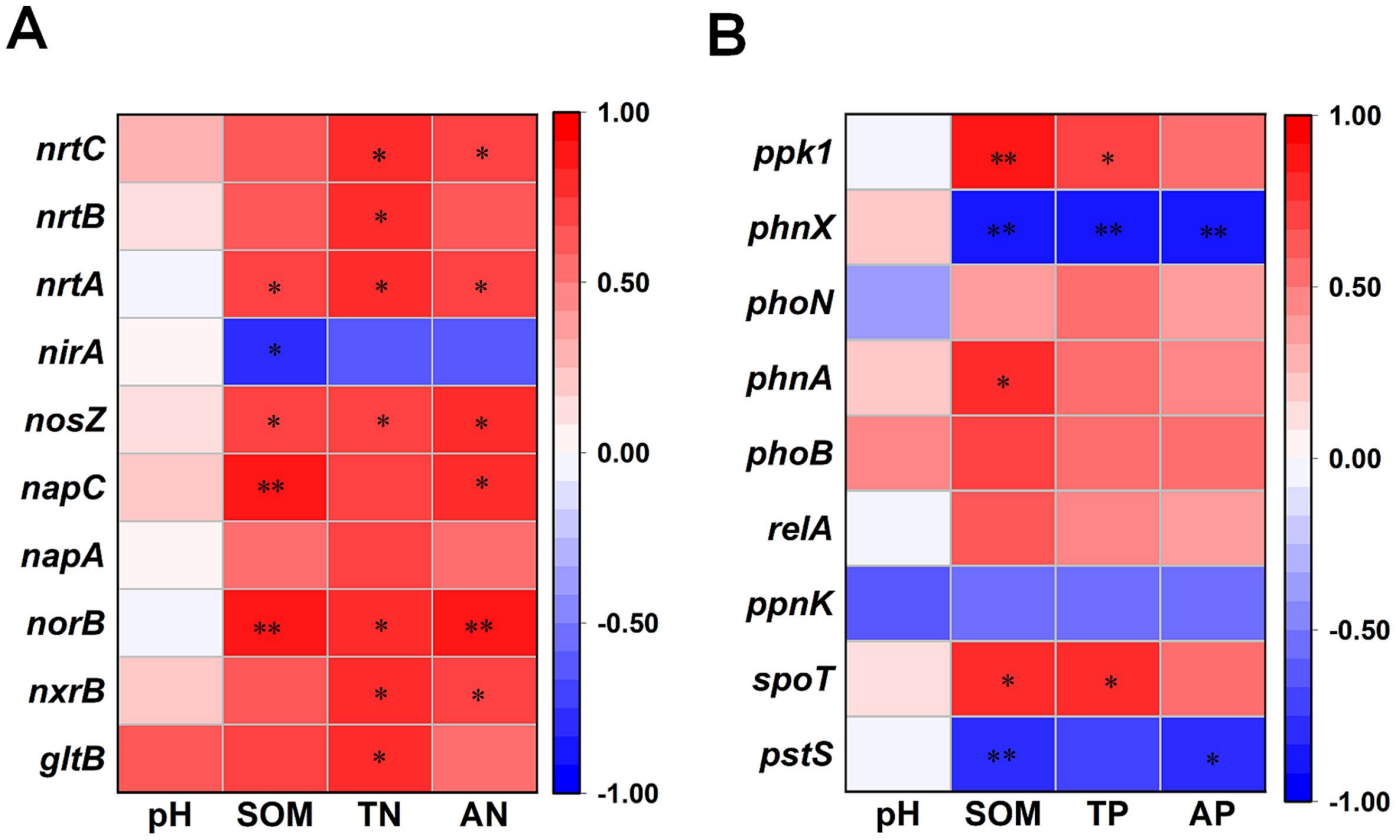
Spearman’s rank correlation heatmaps between soil physicochemical properties and nutrient-cycling functional genes. **(A)** Correlations between soil properties and nitrogen-cycling functional genes; and **(B)** Correlations between soil properties and phosphorus-cycling functional genes. Color intensity represents the magnitude of Spearman’s correlation coefficient (*ρ*), with red indicating positive correlations and blue indicating negative correlations. SOM, soil organic matter; TN, total nitrogen; TP, total phosphorus; AN, alkali-hydrolyzable nitrogen; AP, available phosphorus; CK, no fertilization; OF, organic fertilizer. Significant correlations are presented as asterisks (* *p* < 0.05; ** *p* < 0.01).

The Spearman’s correlation analysis revealed specific associations between N-cycling genes and soil properties (Figure 8A). Soil organic matter was significantly positively correlated with denitrification genes (*norB*, *nosZ*), dissimilatory nitrate reduction genes (*napC*), and nitrogen transporter genes (*nrtA*) (*p* < 0.05), while exhibiting a significant negative correlation with the assimilatory nitrate reduction gene *nirA* (*p* < 0.05). Total N showed significant positive correlations with genes involved in organic N metabolism (*gltB*), nitrification (*nxrB*), denitrification (*norB*, *nosZ*), and nitrogen transport (*nrtA*, *nrtB*, *nrtC*) (*p* < 0.05). Similarly, AN was significantly positively correlated with *nxrB*, *norB*, *napC*, *nosZ*, *nrtA*, and *nrtC* (*p* < 0.05).

#### 3.5.3 Environmental drivers of phosphorus-cycling genes

For P-cycling functional genes, RDA revealed that the first two axes explained 75.32% of the total variance (axis 1: 64.83%; axis 2: 10.49%) (Figure 7B). Furthermore, the results of VPA indicated that pH, SOM, and phosphorus nutrients explained a total of 64.95% of the variation in the abundance of P-cycling functional genes, with each individually explaining 1.03, 4.57, and 9.69%, respectively. Notably, the shared explanatory power of SOM and phosphorus nutrients reached 63.49%.

Spearman’s correlation analysis further elucidated gene-specific responses (Figure 8B). SOM was significantly positively correlated with regulatory genes (*spoT*), phosphonate degradation genes (*phnA*), acid phosphatase genes (*phoN*), and polyphosphate kinase genes (*ppk1*) (*p* < 0.05). In contrast, SOM was significantly negatively correlated with the phosphate transporter gene *pstS* and the phosphonoacetaldehyde hydrolase gene *phnX* (*p* < 0.05). Total P was significantly positively correlated with *spot* and *ppk1* (*p* < 0.05) but negatively correlated with *phnX* (*p* < 0.05). Available P was significantly negatively correlated with *pstS* and *phnX* (*p* < 0.05), suggesting feedback regulation of P acquisition and transport genes under conditions of enhanced P availability.

## 4 Discussion

### 4.1 Organic fertilization restructures soil microbial communities by enriching copiotrophic taxa

Soil microbial communities are central to nutrient cycling and the maintenance of ecosystem functions (Berendsen et al., 2012; Hartmann and Six, 2023). In this study, organic fertilization significantly increased microbial richness without altering overall diversity (Figures 1A,B), a pattern consistent with previous observations Qin et al. (2025). This decoupling of richness and diversity suggests that organic amendments selectively enrich specific microbial taxa adapted to nutrient-rich conditions, rather than broadly stimulating all members of the community (Zhang et al., 2022). Principal coordinate analysis (PCoA) further confirmed a clear shift in overall community structure under organic fertilization (Figure 1C), underscoring the profound impact of nutrient inputs on microbial assembly.

At the phylum level, organic fertilizer application significantly enriched Pseudomonadota and Myxococcota. Members of Pseudomonadota are widely recognized for their involvement in carbon and nitrogen metabolism, playing key roles in nutrient cycling and plant–microbe interactions (Jiang et al., 2019; Kim and Anderson, 2018). Myxococcota, a group of predatory and hydrolytic bacteria, contributes to soil carbon cycling (Wang et al., 2020) and has recently been implicated in promoting nitrogen transformation and utilization (Liu et al., 2024). Their enrichment under organic fertilization likely reflects increased substrate availability and enhanced microbial turnover. Conversely, the relative abundance of Chloroflexota, a phylum typically associated with oligotrophic habitats, was significantly reduced under organic fertilization. Members of Chloroflexota possess high nutrient affinity and are adapted to environments with low resource availability (Huang et al., 2022). Accordingly, their dominance in the unfertilized control (CK) treatment aligns with the low-nutrient conditions. This interpretation is further supported by the significant negative correlations observed between Chloroflexota abundance and multiple soil nutrient parameters, including SOM, TN, TP, and AP (Figure 6B).

At the genus level, organic fertilization significantly enriched *Pseudolabrys* and *Gaiella*. *Pseudolabrys* have been implicated in nitrogen metabolism (Lai et al., 2019), while *Gaiella* is known to participate in nitrogen cycling and the degradation of diverse organic substrates (Chen et al., 2018). Their enrichment under organic fertilization, consistent with previous reports (Qin et al., 2025), suggests that these taxa are key contributors to enhanced nutrient turnover in amended soils.

### 4.2 Organic fertilization enhances the genetic potential for soil nitrogen cycling

Changes in the abundance of functional genes govern the direction and magnitude of soil nitrogen (N) transformations (Chen et al., 2023). In this study, organic nitrogen metabolism constituted the largest proportion of N-cycling functions in rubber plantation soils (Figure 4A), a finding consistent with Huang et al. (2025) and indicative of a strong potential for converting organic N to plant-available ammonium (NH₄^+^-N). Organic fertilizer application significantly enhanced the functional potential of multiple key N-cycling processes, including organic N metabolism, denitrification, N transport, and dissimilatory nitrate reduction to ammonium (DNRA), whereas nitrification remained largely unaffected (Figure 4A). At the gene level, organic fertilization significantly increased the abundance of *gltB*, which encodes glutamate synthase (GOGAT)—a central enzyme in glutamate synthesis and organic N assimilation (Lin et al., 2022). This enrichment suggests an enhanced capacity for microbial N incorporation into biomass under nutrient-rich conditions.

Denitrification and nitrification are critical processes regulating N loss and nitrous oxide (N₂O) emissions. Our results demonstrate that organic fertilization significantly increased the abundance of key denitrification genes, including *norB* (encoding nitric oxide reductase, involved in NO reduction to N₂O) and *nosZ* (encoding nitrous oxide reductase, catalyzing N₂O reduction to N₂). The concurrent enrichment of the nitrification gene *nxrB* (encoding nitrite oxidoreductase, mediating NO₂^−^ oxidation to NO₃^−^) is consistent with previous findings (Liu et al., 2024; Long et al., 2021). Notably, the observed increase in *nosZ* abundance may indicate an enhanced potential for N₂O consumption, potentially mitigating greenhouse gas emissions from fertilized soils (Krause et al., 2017). The increased abundance of *nxrB* may further support downstream processes by ensuring adequate NO₃^−^ supply for DNRA. Correlation analysis revealed that the abundances of *norB* and *nosZ* were positively correlated with soil organic matter (SOM), total nitrogen (TN), and alkaline-hydrolyzable nitrogen (AN) (Figure 8A). These associations suggest that the improved nutrient status under organic fertilization (Table 1) directly contributed to the enrichment of denitrifying communities.

**Figure 9:**
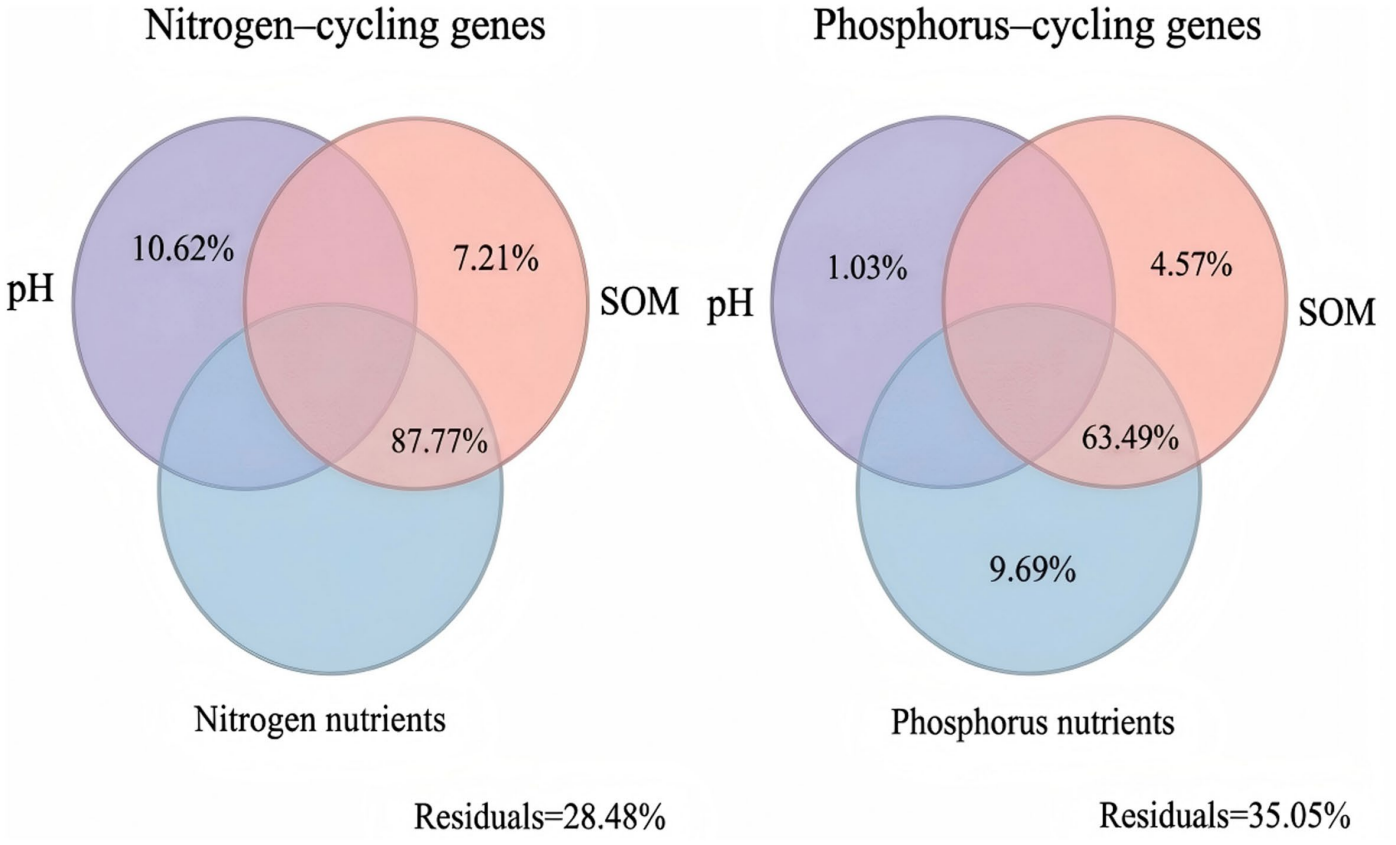
Variance partitioning analysis (VPA) of the contribution of soil physicochemical properties to the variations in nitrogen-cycling genes and the contribution of soil physicochemical properties to the variations in phosphorus-cycling genes.

In the DNRA pathway, *napA* and *nap*C encode periplasmic nitrate reductase components involved in the reduction of NO₃^−^ to NO₂^−^. Their increased abundance under organic fertilization aligns with recent observations (Yang et al., 2024) and may reflect a shift toward N-conserving pathways that retain N as ammonium rather than losing it via denitrification or leaching. Additionally, organic fertilization significantly enhanced the abundances of the high-affinity nitrate transporter genes *nrtA, nrtB,* and *nrtC*, indicating an increased capacity for N acquisition and assimilation under nutrient-enriched conditions. Variation partitioning analysis (VPA) showed that pH, SOM, and nitrogen nutrients collectively explained 71.52% of the variation in the abundance of N-cycling functional genes (Figure 9). These results suggest that variation in N-cycling functional gene abundance in tropical plantation soils was associated with pH, SOM, and nitrogen nutrients, with SOM and nitrogen nutrients showing a particularly large shared explanatory fraction.

### 4.3 Organic fertilization enhances the genetic potential for soil phosphorus cycling

Phosphorus (P) is an essential macronutrient for plants, and its limited bioavailability in soils frequently constrains agricultural productivity, particularly in highly weathered tropical ecosystems (Du et al., 2020). Soil microorganisms play a pivotal role in enhancing P availability through the mineralization of organic P and the solubilization of inorganic P minerals (Bünemann, 2015; Pistocchi et al., 2018). In this study, metagenomic analysis revealed that organic fertilizer (OF) application significantly enriched the genetic potential for organic P mineralization compared to the unfertilized control (CK). Specifically, the abundances of key organic P-mineralizing genes, including phoN (encoding nonspecific acid phosphatase) and *phnA* (encoding phosphonoacetate hydrolase), were significantly elevated in OF-treated soils. These genes have been widely implicated in the hydrolysis of organic P compounds, with their expression directly influencing phosphatase activity and, consequently, soil AP content (Gaiero et al., 2021; Kulakova et al., 2001; Wang et al., 2024). Thus, organic fertilization appears to enhance the microbial capacity for organic P activation, contributing to improved P nutrition. In parallel, organic fertilization significantly enriched genes associated with inorganic P solubilization, a process critical for liberating P from mineral-bound pools (Chai et al., 2021). This finding suggests that organic amendments not only promote the recycling of organic P but also facilitate the dissolution of inorganic P minerals, further augmenting AP availability.

Beyond P mobilization, organic fertilization also influenced intracellular P metabolism. The abundances of *spoT* and *rel*A, genes involved in polyphosphate (poly-P) degradation and the stringent response, were significantly increased under OF treatment. Under P-limited conditions, *spoT* is known to mediate poly-P hydrolysis, mobilizing intracellular P reserves to alleviate P stress (Dai et al., 2024). Conversely, the high-affinity phosphate transporter gene *pstS*, typically upregulated under P deficiency to enhance P scavenging (Hsieh and Wanner, 2010), exhibited a significant negative correlation with soil AP content (Figure 8B). This inverse relationship suggests that as P availability increases with organic fertilization, microbial reliance on high-affinity P uptake systems diminishes—a classic example of feedback regulation at the genetic level.

The *ppk1* gene, encoding polyphosphate kinase, catalyzes the synthesis of poly-P from ATP, serving as both a P reserve and an energy store that enhances microbial fitness in fluctuating environments (Enebe and Babalola, 2021). In this study, *ppk1* abundance was significantly elevated under OF treatment and exhibited strong positive correlations with SOM and TP (Figure 8B). This enrichment suggests that organic fertilization promotes microbial poly-P accumulation, potentially enhancing microbial resilience and P cycling efficiency. Variation partitioning analysis (VPA) showed that pH, SOM, and phosphorus nutrients collectively explained 64.95% of the variation in the abundance of P-cycling functional genes (Figure 9). These results suggest that variation in P-cycling functional gene abundance in tropical plantation soils was associated with pH, SOM, and phosphorus nutrients, with SOM and phosphorus nutrients showing a particularly large shared explanatory fraction.

### 4.4 Research limitations and prospects

This study, using metagenomic sequencing, systematically elucidates how the application of organic fertilizers influences microbial community structure and functional gene expression. Still, some limitations should be acknowledged. Because neither a chemical fertilizer control nor an organic fertilizer application gradient was included, the observed effects cannot yet be unequivocally attributed to the specific influence of organic fertilizer rather than to the general effect of nutrient input. Future studies should therefore incorporate a chemical fertilizer control and multiple levels of organic fertilizer application to more rigorously disentangle nutrient-driven effects from organic fertilizer-specific effects and to identify potential response thresholds under different fertilization intensities. Such expanded experimental designs would strengthen mechanistic interpretation and improve the robustness of conclusions regarding the role of organic fertilizer in reshaping microbial communities and their functional potential.

## 5 Conclusion

This study demonstrates that organic fertilization profoundly reshapes the soil microbiome and enhances the functional potential for nitrogen (N) and phosphorus (P) cycling in tropical rubber plantations. Compared to the unfertilized control, organic fertilizer (OF) application significantly increased soil organic matter (SOM), total nitrogen (TN), total phosphorus (TP), alkali-hydrolyzable nitrogen (AN), and available phosphorus (AP), while selectively enriching copiotrophic microbial taxa such as Pseudomonadota and Myxococcota at the expense of oligotrophic groups like Chloroflexota.

At the functional level, organic fertilization enhanced multiple N-cycling pathways, including organic N metabolism (*gltB*), N transport (*nrtA, nrtB, nrtC*), denitrification (*norB, nosZ*), nitrification (*nxrB*), and dissimilatory nitrate reduction (*napA, napC*). In the P cycle, organic fertilization alleviated P limitation, as evidenced by the downregulation of the high-affinity phosphate transporter gene *pstS* and the concurrent upregulation of genes governing organic P mineralization (*phnA, phoN*), regulation (*phoB*), polyphosphate synthesis (*ppk1*), and polyphosphate degradation (*spoT, rel*A). These shifts collectively enhanced the soil’s capacity for P mobilization and storage. Furthermore, the study identified that pH, SOM, and nitrogen/phosphorus nutrients jointly explained most of the variation in the abundance of N-and P-cycling functional genes (71.52 and 64.95%, respectively), underscoring their central roles in driving microbial nutrient transformation.

Together, these findings provide a mechanistic understanding of how organic fertilization regulates soil N and P cycling from a microbial-ecological perspective, offering a theoretical foundation for developing sustainable nutrient management strategies in rubber agroecosystems.

## Data Availability

The datasets presented in this study can be found in online repositories. The names of the repository/repositories and accession number(s) can be found in the article/supplementary material.

## Footnotes

1
http://www.genome.jp/kegg/
